# The Probable Role of Cytomegalovirus in Acute Myocardial Infarction

**DOI:** 10.5812/jjm.9253

**Published:** 2014-03-01

**Authors:** Morteza Izadi, Mohammad Mahdi Zamani, Nastaran Sabetkish, Hassan Abolhassani, Seyed Hassan Saadat, Saeed Taheri, Hossein Dabiri

**Affiliations:** 1 Health Research Center, Baqiyatallah University of Medical Sciences, Tehran, IR Iran; 2 Students’ Scientific Research Center, Tehran University of Medical Sciences, Tehran, IR Iran; 3 Behavioral Sciences Research Center, Baqiyatallah University of Medical Sciences, Tehran, IR Iran; 4 Department of Medical Microbiology, Faculty of Medicine, Shahid Beheshti University of Medical Sciences, Tehran, IR Iran

**Keywords:** Atherosclerosis, Cadaver, *Chlamydia pneumonia*, Coronary Artery Disease, Cytomegalovirus, *Helicobacter pylori*

## Abstract

**Background:**

Coronary artery disease (CAD) is the most common cause of death worldwide and many studies have been performed on reduction of its prevalence.

**Objectives:**

This case control study was designed to investigate the presence of Cytomegaloviruses, *Chlamydia pneumoniae* and *Helicobacter pylori* in atherosclerotic plaques of cadaveric coronary endothelium of patients with and without acute myocardial infarction.

**Patients and Methods:**

Sixty cadavers in two equal groups were analyzed. Acute myocardial infarction group included cadavers with acute myocardial infarction and atherosclerotic plaque. The non- acute myocardial infarction group included those with innocent atherosclerotic plaques in autopsy, expired due to other causes. Specimens from coronary vessels’ atherosclerotic plaque were taken and studied by polymerase chain reaction for Cytomegaloviruses, *C. pneumoniae* and *H. pylori*.

**Results:**

Cadavers of 26 males and 34 females underwent autopsy procedures. Their mean age at the time of death was 48.17 ± 18.74 years. Unknown causes (20%), hanging (20%), head trauma (16.7%) and multiple traumas (13.3%) were the most common causes of death in the non- acute myocardial infarction group. PCR test results were negative for *C. pneumoniae* and *H. pylori *in all cadavers of both groups. Nine cadavers from the acute myocardial infarction group and one from the non- acute myocardial infarction group showed positive PCR results for Cytomegaloviruses (30% and 3.33%, respectively). There was a significant difference between the two groups regarding Cytomegaloviruses positivity in coronary artery plaques (P < 0.01, odd ratio: 12.42, 95% CI: 10.46 to 15.73).

**Conclusions:**

A significant proportion of coronary atherosclerotic plaques in cadavers with confirmed acute myocardial infarction were detected to be infected with Cytomegaloviruses while no infections of *C. pneumoniae* and *H. pylori *were detected.Coronary artery disease (CAD) is the most common cause of death worldwide and many studies have been performed on reduction of its prevalence.

## 1. Background

Cardiac and cerebral vessel diseases are the main causes of death in developing countries by pathogenesis of atherosclerosis. The total direct and indirect costs of cardiovascular disease (CVD) and stroke in the United States is estimated to be $503.2 billion in 2010; which is higher than for any other diagnostic group (1). Coronary artery disease (CAD), as the major category of CVD, is under evaluation for more novel preventable risk factors (2). Along with the known risk factors of CAD such as hypertension and diabetes mellitus, certain bacterial and viral infections, as present suspicious risk factors, are being considered as definite risk factors. Recent studies on myocardial infarction, have shown direct and indirect biological or biochemical effects of *Helicobacter pylori*, *Chlamydia pneumoniae* and Cytomegalovirus on atherosclerosis process (3-5) as well as acute myocardial infarction (6). 

Suggested mechanisms are macrophage activation, endothelial cell injury and lipid retention in the atherosclerotic plaques (7, 8). In an experimental study in 1979, typical atherosclerosis was developed with birds contaminated by Gallid herpesvirus (9). This study was a clue for more detailed evaluations on the possible effects of Cytomegaloviruses (a member of *Herpesviridae* family) on atherosclerosis. The association between *C. pneumoniae* and atherosclerosis in comparison with other agents has been investigated in previous studies. Several evidences have indicated the role of *C. pneumoniae* in atherosclerosis development by using seroepidemiological, histological and experimental examinations (10, 11).

Several studies were performed to investigate the correlation between *H. pylori *and atherosclerosis (12, 13) As a new approach toward *H. pylori*, high levels of IgG, produced against *H. pylori *were documented in patients with acute coronary syndrome, the results of which confirmed that in acute coronary syndrome, the level of *H. pylori *-IgG augments significantly (14). Other studies have shown no difference between IgG level or polymerase chain reaction (PCR) of *H. pylori *in CAD patients compared to healthy controls (15, 16). Some studies confirmed the correlation between acute myocardial infarction and the presence of Cytomegaloviruses and *C. pneumoniae* (serology or PCR) (17, 18) all performed on living patients. Consequently performing a similar study on cadaveric vessels with atherosclerotic plaques (with or without acute myocardial infarction) for the purpose of evaluating the presence of infective agents, may guide us to prove or disprove the correlation of common bacterial and viral infections with CAD. Cadavers’ autopsy is the best choice for performing more detailed investigations to confirm associations between acute myocardial infarction and probable risk factors.

## 2. Objectives

The aim of this study was to assay the presence of Cytomegaloviruses, *C. pneumoniae* and *H. pylori *in endothelium of cadavers whose death was due to either acute myocardial infarction or non- acute myocardial infarction causes.

## 3. Patients and Methods

### 3.1. Study Design

In this case control study, cadavers who were transferred to Kahrizak Forensic Center (the largest forensic center of Tehran, Iran), were investigated from July to December 2010. Cadavers had undergone routine autopsies because of sudden death in the previous 24 hours. Sixty cadavers possessing atherosclerotic plaque in autopsies (with the size more than 1 × 1 mm) were included in the present study. All subjects’ parents/guardians signed a written informed consent before the autopsy. Institutional Review Board approval was granted by the local Research Ethic Committee of Baqiyatallah University of Medical Sciences. All atherosclerotic plaques were analyzed and confirmed by two pathologists.

Cadavers, who had medical evidences of positive Cytomegaloviruses, *C. pneumoniae* or *H. pylori *in their medical records, for their last year of life, or had any medical evidences of immunodeficiency, were excluded from this study. Thirty cadavers with confirmed history of acute myocardial infarction were selected for the acute myocardial infarction group and thirty cadavers not expiring due to acute myocardial infarction, were categorized in to the non- acute myocardial infarction group. History of acute myocardial infarction was defined as finding at least one of the following criteria (19) from cadavers’ medical records: I) Detection of rise and/or fall of the troponin I enzyme to the upper limit of the normal range together with evidence of myocardial ischaemia accompanied with at least one of the symptoms of ischaemia or electrocardiogram pattern changes indicative of new ischaemia (new ST-T changes or new left bundle branch block [LBBB]); development of pathological Q waves. II) Sudden, unexpected cardiac death, involving cardiac arrest, often with symptoms suggestive of myocardial ischaemia and accompanied with presumably new ST elevation, or new LBBB, and/or evidence of fresh thrombus at autopsy. III) Pathological findings of acute myocardial infarction.

All cadavers were analyzed for the mentioned criteria of acute myocardial infarction. Cadavers, not passing any of the criteria mentioned above, were included in the non- acute myocardial infarction group. If there was no other defined death causes according to routine laboratory and pathological analysis of the forensic center, ‘unknown’ death cause was recorded for that cadaver.

### 3.2. Specimen Collection

Tissue specimens were dissected in the forensic center under sterile conditions by a cardiologist. Coronary artery specimens (containing plaques) were divided into two similar macroscopic parts. One part was transmitted to the pathology center and the second part was transferred in micro centrifuge tubes and kept at -20˚C without any binding buffer until processing.

### 3.3. Sample Preparation and Primers’ Design for PCR

DNA was extracted from endarterectomy specimens by utilization of QIAamp tissue mini-kit (Qiagen Inc, Valencia, USA). *H. pylori *gene primers used for PCR were C97 (forward): 5'-GCTATGACGGGTATCC-3' and C98 (reverse): 5'-GATTTTACCCCTACACCA-3', which amplify a 398 base-pair (bp) fragment of the 16S rRNA gene. All PCR mixtures were performed in a total volume of 25 µL, containing 10 mL PCR buffer, 500 nM of each primer, 2 mM MgCl_2_; 200 µM of each dNTP, 1.5 U Taq DNA polymerase and 200 ng DNA sample. Initial denaturation of mixture was performed for 5 minutes at 94˚C, followed by 30 cycles of denaturation at 93˚C for 1 minute. After the final extension, PCR products were visualized by electrophoresis in 1.2% agarose gel, stained with ethidium bromide and examined under UV illumination (20).

For *C. pneumoniae*, primers which were used for PCR were HL1 (5'-GTTGTTCATGAAGGCCTACT-3') and HR1 (5'-TGCATAACCTACGGTGTGTT-3'), which amplified a 437 bp fragment of the Pstl cloned gene (21). PCR products were visualized by electrophoresis on 1.5% agarose gel, stained with ethidium bromide and examined under UV illumination. Primers from the gB region of the Cytomegaloviruses genome were utilized for PCR of Cytomegaloviruses. The forward and reverse primers were 5ˊ-CGGTGGAGATACTGCTGAGGTC-3ˊ and 5ˊ-CAAGGTGCTGCGTGATATGAAG-3ˊ, respectively. The reaction mixture of the PCR contained a total volume of 50 µL, containing 75 mM Tris-HCL (pH = 9), 1.5 mM MgCl_2_, 50 mM KCl, 20 mM (NH_4_)_2_SO_4_, 50 µL of each one of the deoxynucleoside and triphosphatase solutions, 20 pM of primers (gB1 and gB2) and 1 µg of DNA obtained from tissue.

The reaction mixture was first incubated at 94˚C for 3 minutes, followed by 40 cycles of 94˚C for 30 seconds, 55˚C for 30 seconds, 72˚C for 30 seconds and finally for 3 minutes at 72˚C. The PCR products were subjected to electrophoresis on a 2% agarose gel and 257 bp amplicons were visualized by ultraviolet light after ethidium bromide staining. Each Cytomegaloviruses PCR assay included a positive control with human Cytomegalovirus (HCMV) AD169 DNA and a negative control containing no template (only distilled water).

### 3.4. Statistical Analysis

Data are expressed as mean ± SD, odds ratio and 95% confidence interval (95% CI). The Statistical Package of Social Science version 16.0 (SPSS, Chicago, Illinois, USA) was used for data analysis. P ≤ 0.01 was considered statistically significant. For the age comparison between the two groups, independent samples t-test was used while the Chi-Square test was used for comparison of positivity of infection between the groups. Mantel Hansel test (Odds ratio: ORMH) was used to eliminate confounder factors.

## 4. Results

Autopsy was done on 26 males and 34 female’ cadavers. Similar number of females and males were placed in both groups, 17 females vs. 13 males. Therefore there was no significant gender difference between the groups. Age range was 18 to 83 years. The mean age of subjects at the time of death was 48.17 ± 18.74 years for all of them and; 59.50 ± 15.06 and 36.83 ± 14.90 years in acute myocardial infarction and non- acute myocardial infarction group, respectively. There was a significant difference between the mean ages of the two groups (P < 0.01). Most common death causes in the non- acute myocardial infarction group were unknown (20%), hanging (20%), head trauma (16.7%) and multiple traumas (13.3%). Death causes of non- acute myocardial infarction group are presented in Table 1. 

Nine cadavers from the acute myocardial infarction group were Cytomegaloviruses positive (30.0%), while non- acute myocardial infarction group had only one Cytomegaloviruses positive (3.33%) cadaver. There was a significant difference between the two groups regarding Cytomegaloviruses positivity in coronary artery plaques (P < 0.01, odd ratio: 12.42, 95% CI: 10.46 to 15.73). Table 2 visualizes the Cytomegaloviruses PCR results in age categories for deconfounding age effects. ORMH PCR results for *H. pylori *and *C. pneumoniae* were negative in all cadavers of both acute myocardial infarction and non- acute myocardial infarction groups.

**Table 1.** Death Causes of Cadavers in Non-acute Myocardial Infarction (non- acute myocardial infarction) Group

Death Cause	Unknown	Hanging	Head Trauma	Multiple Traumas	Internal Diseases	Animal Bites	Burning	Cerebral Vessel Disease	Pneumonia
**Percentage**	20	20	16.7	16.7	6.7	6.7	6.7	3.3	3.3

**Table 2.** The Polymerase Chain Reaction Results of Cytomegalovirus in Age Categories

Age Category, y	No. (%)	Cytomegaloviruses		P Value
		Negative	Positive	
**16-35**				
Cases	2 (3.33)	2 (3.33)	0	0.04
Controls	17 (28.33)	16 (26.66)	1 (1.67)	
**36-60**				
Cases	13 (21.66)	9 (15)	4 (6.67)	0.000 ^a^
Controls	11 (18.33)	11 (18.33)	0	
**≥ 61**				
Cases	15 (25)	10 (16.66)	5 (8.33)	0.001 ^a^
Controls	2 (3.33)	2 (3.33)	0	0

^a^ P ≤ 0.01.

## 5. Discussion

Chronic infections and atherosclerosis had uncertain associations for years (22). *H. pylori *was approved as a possible risk factor for ischemic vascular disease in 1998, (3), and *C. pneumoniae* was known as a suspicious agent in atherosclerosis process (4, 23). In a study executed in the United States during 1996, Cytomegaloviruses preventive therapy with ganciclovir showed effectiveness in prevention of restenosis and atherosclerosis in transplanted organs (24). In a few studies, presence of Cytomegaloviruses IE (immediate early) antigen and Cytomegaloviruses DNA in the vessel wall was associated with the pathological process of atherosclerosis formation (25).

Furthermore, Cytomegaloviruses positivity was introduced as a result of atherosclerosis plaque formation; because active Cytomegaloviruses infection was present in a greater proportion of CAD patients rather than healthy controls indicating that CAD patients are more susceptible of Cytomegaloviruses (26). The most ideal study is the measurement of Cytomegaloviruses DNA and serology criteria before and after acute myocardial infarction with regular intervals, yet as it cannot predict the acute myocardial infarction incidence, this study can strongly confirm the need for further evaluations with periodic laboratory studies of CVD patients until their probable acute myocardial infarction incidence is determined. Our study was designed to determine whether chronic infections make a poor prognosis for atherosclerosis plaques or immunological changes after acute myocardial infarction due to atherosclerosis plaques make a good environment for common bacterial and viral agents.

Investigation of *H. pylori*, *C. pneumoniae* and Cytomegaloviruses in coronary artery specimens of cadavers with atherosclerotic plaque, ding in 24 hours with and without acute myocardial infarction, could answer this question to some extent. Our study on cadavers has shown significant Cytomegaloviruses positivity in cadavers expiring due to acute myocardial infarction (P < 0.01) while *H. pylori *and *C. pneumoniae* positivity were not detected. Although cadavers do not have immunological blood changes (the absence of increased cytokines serum concentration), yet in our study cadavers showed higher rates of Cytomegaloviruses positivity in coronary arteries with atherosclerotic plaques. These results suggest that Cytomegaloviruses infection can affect atherosclerosis prognosis proposing that chronic infections such as Cytomegaloviruses can worsen atherosclerotic plaques resulting in CAD.

Yi et al. investigated the presence of Cytomegaloviruses and its antigens in internal carotid arteries of patients with cerebral vascular attacks in a Chinese population during 2008, showing the significantly higher presence of Cytomegaloviruses DNA, in the case group compared to those without cerebral vascular attacks (25). During 2009 Xenaki et al. reported no significant difference between specimens of coronary arteries with atherosclerotic plaques and specimens of normal vessels regarding Cytomegaloviruses positivity (27). However, the atherosclerotic and normal samples were extracted from the same subjects. On the other hand, Xenaki’s study confirmed that Cytomegaloviruses infection may not result in atherosclerosis and that atherosclerotic plaque formation did not increase the incidence of Cytomegaloviruses infection; the latter was similar to that found in the present study. Additionally this study did not confirm the presence of *C. pneumoniae* and *H. pylori *in neither acute myocardial infarction nor non- acute myocardial infarction group.

This could suggest that there is no association between these two bacteria and progression of atherosclerotic plaque mortality due to acute myocardial infarction, while in a study by same authors in 2009 (28), *C. pneumoniae* was detected in high proportions in atherosclerotic plaques compared to the control group supporting the role of *C. pneumoniae* in atherosclerotic plaque formation. Ozdogru et al. (16) published a study on *H. pylori *-IgG in three groups of angiographic proven CAD patients including 163 myocardial infarctions, 106 unstable anginas and 84 stable anginas, compared to 163 controls with normal coronary arteries. Ozdogru proposed similar seropositivity rates for *H. pylori *in the control and patient groups and the results were approved in the present study stating no differences were detected in *H. pylori *DNA between the two groups of cadavers. However this study is limited to dead cases, the result may be different in alive cases.

One potential criticism may arise over this study and that is the significant age difference between our groups. About Cytomegaloviruses epidemiology in a study from Finland, the seroprevalence rates were 47% in 10 to 12 year-olds, 68% in 15 to 35 year-olds and 81% among 36 to 60 year-olds (29). According to this age distribution, we did not have any significant difference among age categories mentioned above in our two groups (Table 2). In a US population based study, Cytomegaloviruses seroprevalence increased from 36% in 6 to 11 year-olds to 91% in those aged > 80 years (30). We had one cadaver from the acute myocardial infarction group, older than 80 years old, with negative Cytomegaloviruses PCR test result (*H. pylori *and *C. pneumoniae* were also negative).

In the mentioned US study, other factors associated with Cytomegaloviruses seropositivity were female sex, foreign birthplace, low household income, household crowding, and low household education (31); but in this study, the gender difference between acute myocardial infarction and non- acute myocardial infarction group was not significant. Dowd et al. in 2009 reported that socioeconomic inequality affected early and middle age seroprevalence of Cytomegaloviruses infection in a US population. It is substantial to evaluate these variables in future similar studies on cadavers.

It was conclude that Cytomegaloviruses chronic infection may have a definite role in prognosis of atherosclerotic plaques but it does not have any effects in atherosclerotic plaques formation, so attempts for eradication of Cytomegaloviruses in CAD proven patients may decrease the prevalence of acute myocardial infarction and improve their prognosis.
